# Bretschneider (Custodiol®) and St. Thomas 2 Cardioplegia Solution in Mitral Valve Repair via Anterolateral Right Thoracotomy: A Propensity-Modelled Comparison

**DOI:** 10.1155/2019/5648051

**Published:** 2019-12-04

**Authors:** Constantin Mork, Luca Koechlin, Thibault Schaeffer, Lena Schoemig, Urs Zenklusen, Brigitta Gahl, Oliver Reuthebuch, Friedrich S. Eckstein, Martin T. R. Grapow

**Affiliations:** Department of Cardiac Surgery, University Hospital Basel, Basel, Switzerland

## Abstract

**Background:**

Single-dose cardioplegia is preferred in minimal invasive mitral valve surgery to maintain the adjustment of the operative site without change of preset visualization. The aim of our study was to compare two widely used crystalloid cardioplegias Bretschneider (Custodiol®) versus St. Thomas 2 in patients who underwent mitral valve repair via small anterolateral right thoracotomy.

**Material and Methods:**

From May 2012 until February 2019, 184 isolated mitral valve procedures for mitral valve repair via anterolateral right thoracotomy were performed using Bretschneider (Custodiol®) cardioplegia (*n* = 123) or St. Thomas (*n* = 61). Primary efficacy endpoint was peak postoperative high-sensitivity cardiac troponin (hs-cTnT) during hospitalization. Secondary endpoints were peak creatine kinase-muscle brain type (CK-MB) and creatine kinase (CK) as well as safety outcomes. We used inverse probability of treatment weighting (IPTW) in order to adjust for confounding by indication.

**Results:**

Peak hs-cTnT was higher after use of Bretschneider (Custodiol®) (geometric mean 716 mg/L, 95% confidence interval (CI) 605-847 mg/L) vs. St. Thomas 2 (561 mg/L, CI 467-674 mg/L, *p* = 0.047). Peak CK-MB (geometric mean after Bretschneider (Custodiol®): 40 *μ*g/L, CI 35-46, St. Thomas 2: 33 *μ*g/L, CI 27-41, *p* = 0.295) and CK (geometric mean after Bretschneider (Custodiol®): 1370 U/L, CI 1222-1536, St. Thomas 2: 1152 U/L, CI 972-1366, *p* = 0.037) showed the same pattern. We did not see any difference with respect to postoperative complications between treatment groups after IPTW.

**Conclusion:**

Use of St. Thomas 2 cardioplegia was associated with lower postoperative peak levels of all cardiac markers that reflect cardiac ischemia such as hs-cTnT, CK, and CK-MB as compared to Bretschneider (Custodiol®) in propensity-weighted treatment groups.

## 1. Introduction

Mitral regurgitation is the second most common valve disease and a common indication for valve surgery in Europe. Therefore, mitral valve repair is the gold standard for the correction of severe mitral regurgitation and recommended (class I) by current ESC/EACTS guidelines for the management of valvular heart disease [1]. Minimal invasive mitral valve surgery via a minithoracotomy has become a preferred surgical approach to treat mitral valve pathologies. This procedure is less invasive; hence, it has a lower risk of severe wound complications and is cosmetically more attractive to patients, while being technically more demanding for the cardiac surgeon. Several studies have shown the benefit of minimal invasive cardiac surgery due to decreased blood loss and shorter ICU stay [2]. Anesthesia management has improved along with postoperative care and as a consequence has the surgical outcome. Due to the increasing complexity of patients and limited surgical access, myocardial protection strategies became the focus of surgical attention. During the procedure of mitral valve repair, there is no doubt that optimal myocardial protection plays a key role in achieving a successful outcome and in minimizing myocardial damage. However, there is an ongoing discussion about the optimal cardioplegic solution for myocardial preservation. Several smaller studies have shown the benefit of using crystalloid cardioplegia in cardiac surgery [3–9]. The striking advantage of crystalloid solutions is the longevity of duration of a single dose with avoidance of several adjustments of the operative site, which is necessary in other cardioplegic formulas with repetitive administrations. To assess possible differences among crystalloid cardioplegia solutions, we included patients in whom single-dose antegrade crystalloid cardioplegia with either Bretschneider (Custodiol®) or St. Thomas 2 was used. Both are very appealing for minimally invasive cardiac surgery due to their single-dose application and secure cardioprotective arrest for up to two hours.

The aim of our study was to investigate whether St. Thomas 2 provides superior myocardial protection and decreases myocardial damage compared to Bretschneider (Custodiol®) in patients undergoing full endoscopic minimal invasive anterolateral right minithoracotomy.

## 2. Methods

### 2.1. Ethical Approval

The local ethical committee (EKNZ BASEC Req-2019-00070) approved the study protocol, which is in accordance with the principles of the declaration of Helsinki. The ethical committee has waived the need to obtain informed consent.

The trial was registered at the ClinicalTrials.gov (ID NCT03818113). The authors designed the study, gathered and analyzed the data, vouched for the data and analysis, wrote the paper, and decided to publish.

### 2.2. Study Population

We included patients who underwent isolated mitral valve surgery using an anterolateral minithoracotomy with femoral installation of extracorporeal circulation (ECC) in the department of cardiac surgery at the University Hospital Basel from May 2012 until February 2019. Patients received either St. Thomas 2 or Bretschneider (Custodiol®) cardioplegia as a single dose. Standard protocol for Bretschneider (Custodiol®) included antegrade delivery of 20 mL/kg and for St. Thomas 2 30 mL/kg within 6 minutes at 4°C perfusion temperature. We excluded patients who had known coronary artery disease, mitral valve replacement, second cross-clamping, and combined cardiac procedures.

A group of 123 patients, who received Bretschneider (Custodiol©) cardioplegia, was compared to 61 patients with St. Thomas 2 cardioplegia. Given the observational origin of the data, we used IPTW to adjust for confounding by indication, including age, female gender, logistic EuroSCORE, and hypertension as covariates into the propensity model. We trimmed the tails of the propensity score, as the corresponding records are suspicious of residual confounding, and calculated standardized differences to assess comparability of treatment groups after IPTW.

### 2.3. Surgical Technique

In brief, minimally invasive mitral valve repair is performed through a right anterolateral thoracotomy with a periareolar incision, usually entering the thoracic cavity through the fourth intercostal space. Usually, the left groin is used for venous and arterial femoral cannulation for the cardiopulmonary bypass. For the first two years, we used the Chitwood clamp; later, we switched to the Glauber clamp for aortic cross-clamping. Either St. Thomas 2 or Bretschneider (Custodiol®) cardioplegia as a single dose is administered. In all our cases, mitral valve repair with leaflet resection and/or implantation of artificial chordae in combination with ring annuloplasty is exclusively performed. Every patient is monitored by transesophageal echocardiography, which is used perioperatively to evaluate the surgical results.

### 2.4. Outcomes

Primary efficacy outcome was the highest postoperative hs-cTnT level measured before discharge; secondary outcomes were peak CK-MB and CK and the first postoperative hs-cTnT, CK-MB, and CK, which have been measured at the first day after surgery at 6 am. Safety outcomes were postoperative complications such as myocardial infarction, MACCE, and need for defibrillation at the end of surgery. Operative mortality was defined as death during surgery. Postoperative stroke was defined as a new, permanent neurological disability or deficit. Atrial fibrillation was defined as a new and permanent postoperative atrial fibrillation during hospitalization. Length of hospital stay was defined as one day prior to the procedure until discharge.

### 2.5. Statistical Analysis

We conducted an IPTW analysis and included age, female gender, EuroSCORE 2, hypertension, last preoperative creatinine, ejection fraction (EF), prior myocardial infarction (MI), and preoperative stroke as covariates into the propensity model. We trimmed the tails of the propensity score distribution. Differences between the treatment groups (Bretschneider (Custodiol®) and St. Thomas cardioplegia 2) after IPTW were expressed as standardized differences to assess the difference independent of the number of observations. We considered an absolute standardized difference above 0.1 to be indicative for a possibly meaningful difference. Given the skewed distribution of hs-cTnT, CK-MB, and CK, these variables were analyzed as geometric mean with confidence interval which was back transformed from the logarithmic scale. To investigate the impact of cardioplegic solution on these cardiac markers, we used IPT-weighted Poisson regression with robust standard errors. We included size of ring for annuloplasty as covariate into the model as a first sensitivity analysis. As a second sensitivity analysis, we included year of surgery as fractional polynomial covariate into the model to adjust for the impact of changes in surgical details that might have happened within 7 years of patients' enrolment.

Normally distributed continuous data were reported as mean ± standard deviation, and comparisons were made using linear regression. Nonnormally distributed continuous variables were analyzed the same way as cardiac markers. Categorical data are reported as numbers with percentage and compared using logistic regression. Confidence intervals and *p* values are two-sided; a *p* value below 0.05 is considered significant. All analyses were done by a biostatistician (BG) using Stata 15 (StataCorp, Texas).

## 3. Results

### 3.1. Baseline Characteristics

From January 2009 to February 2019, 871 patients underwent 904 mitral valve operations at our hospital. In 695 patients who received mitral valve repair, anterolateral thoracotomy was chosen as surgical access in 269 patients. 184 of which met the inclusion criteria of this study.

The median age of the patients in the group of Bretschneider (Custodiol®) was 63 years (SD = ±11.8) and in the group of St. Thomas 2 64 years (SD = ±10.2), respectively (*p* = 0.48). Male gender was predominant in both groups (Bretschneider (Custodiol®) *n* = 64% vs. St. Thomas 2 *n* = 66%). Before IPTW, groups were comparable with respect to hemodynamic profiles and all other baseline characteristics except hypertension which was more frequent in patients treated with Bretschneider (Custodiol®). After IPTW, treatment groups were similar with respect to patient characteristics and mitral valve pathology (Tables 1 and 2).

### 3.2. Surgical Results

Duration of operation and aortic clamping time were similar in both groups (208 min vs. 207 min and 90 min vs. 88 min, respectively). There was no statistical difference in perfusion time. Defibrillation was more frequently used in patients receiving Bretschneider (Custodiol®) cardioplegia (50%) vs. St. Thomas 2 (36%). Both cardioplegias were given as a single-shot antegrade. Neither of the two groups received a second injection of Bretschneider (Custodiol®) or St. Thomas 2 cardioplegia. The hemodynamic profiles of the matched patients were comparable in both groups. Both groups had predominantly a valve annuloplasty.

### 3.3. Postoperative Data

The in-hospital outcomes and complications are summarized in Table 3 after IPTW. In terms of in-hospital mortality, there was no significant difference between the two groups (Bretschneider (Custodiol®) *n* = 1 (0.7%) vs. St. Thomas 2 *n* = 0 (0%); *p* = 0.484). Although there were three cases (*n* = 2.4%) of myocardial infarction in the group of Bretschneider (Custodiol®), these were without statistical significance in comparison with the group of St. Thomas 2 (*n* = 0%, *p* = 0.22). Moreover, we did not find any statistically significant difference in the stay of the hospital length in days and for atrial fibrillation at discharge (Bretschneider (Custodiol®) *n* = 32 (28.5%) vs. St. Thomas *n* = 15 (27.7%); *p* = 0.918).

### 3.4. Efficacy Outcome

Postoperative biomarkers including creatine, CRP, GFR, potassium, hemoglobin, leucocytes, sodium, hs-cTnT, CK, and CK-MB are shown in Table 4.

After IPTW, our primary outcome peak hs-cTnT was higher after use of Bretschneider (median = 716 ng/L) as compared to St. Thomas 2 (median = 561 ng/L; *p* = 0.0047). The first hs-cTnT and CK showed the same pattern.

There was a statically significant difference in the first CRP (Bretschneider (Custodiol®) mean = 51.1 mg/L vs. St. Thomas 2 mean = 64.2 mg/L; *p* = 0.005). Additionally, the first potassium was significantly lower in the group of Bretschneider (Custodiol®) (*p* = 0.017) but in the normal range of lab analysis. All cardiac markers measured in blood were higher in the group of Bretschneider (Custodiol®). These results were robust after further adjustment for size of ring (*p* = 0.027) but less pronounced after adjustment for year of enrolment (*p* = 0.054).

## 4. Discussion

This study has shown the benefit of St. Thomas 2 cardioplegia regarding the postoperative cardiac markers in minimally invasive mitral valve repair.

The purpose of cardioplegia is to cause myocardial arrest, to support normal physiology during ischemia, and to decrease the metabolism. Bretschneider (Custodiol®) and St. Thomas 2 are appealing cardioplegic agents for minimal invasive cardiac surgery. Both are given as a single dose and offer myocardial protection for up to 2 hours. On the one hand, Bretschneider (Custodiol®) cardioplegia is an intracellular crystalloid cardioplegic solution with low concentration of calcium and sodium. On the other hand, St. Thomas 2 cardioplegia is an extracellular cardioplegic solution, which is based on potassium and magnesium. Few studies compare the effect of either Bretschneider (Custodiol®) or St. Thomas 2 with other established cardioplegic solutions, but they have never been compared directly [10–13].

This retrospective analysis focused on patients with a minimally invasive mitral valve repair via an anterolateral right thoracotomy. Patients receiving a single dose of Bretschneider (Custodiol®) were compared with patients receiving St. Thomas 2 cardioplegia regarding the postoperative cardiac markers such as hs-cTnT, CK, and CK-MB. Our primary endpoint was the hs-cTnT postoperatively, and the secondary endpoints were the CK, CK-MB, and safety outcomes. The patients' characteristics were similar in both groups.

We measured significantly lower cardiac markers postoperatively in patients receiving St. Thomas 2 cardioplegia in mitral valve repair via an anterolateral thoracotomy. Since the surgeons had the same experience, the duration of the operation, the cross-clamping time, and the perfusion time were statistically not significant. The number of perioperative defibrillation was statistically not significant, but there is a tendency in the group of St. Thomas 2, which needed fewer defibrillations (*n* = 20 (37%) as compared to Bretschneider (Custodiol®) *n* = 56 (50%); *p* = 0.130). There were no statistically significant differences in the postoperative outcomes. However, all postoperative cardiac markers were elevated in the group of Bretschneider (Custodiol®). In particular, the maximum values of hs-cTnT and CK were significantly increased compared to the group with St. Thomas 2 cardioplegia (hs-cTnT: median 716 ng/L vs. 561 ng/L; *p* = 0.047; CK: median 1370 U/L vs. 1152 U/L; *p* = 0.037).

There are some limitations in our study: First, this is a single-center observational study. The sample size is relatively small, and there is a mismatch in the number of patients in these two groups. After IPTW, 113 patients were treated with Bretschneider (Custodiol®) as cardioplegic solution versus 55 patients who were given St. Thomas 2 cardioplegic solution for myocardial protection during cardiac surgery. Second, measurement of the postoperative biomarkers was not according to a specific time. Time frame for the first postoperative hs-cTnT, CK, and CK-MB was within the first 36 hours after the cardiac surgery. The maximum values of high-sensitive troponin, creatine kinase, and creatine kinase-myocardial type were defined/measured as maximum during hospitalization. Additionally, in comparison to hs-cTnT and CK-MB, CK has a low specificity as a biomarker for myocardial damage. Concerning St. Thomas 2, it seems unusual to use it as a single shot, but we never had to reperfuse St. Thomas 2 cardioplegia due to cardiac activity while performing mitral valve repair.

## 5. Conclusion

It is important to understand that such a complex operation like mitral valve repair with Bretschneider (Custodiol®) or St. Thomas 2 cardioplegia should be done without having interruption or complex changes in the position of the heart. Single-dose antegrade cold Bretschneider (Custodiol®) and St. Thomas 2 in elective valve surgery are both effective in protecting the myocardium. Both cardioplegia strategies had a similar cross-clamp time. All postoperative cardiac markers were higher in the group of Bretschneider (Custodiol®) cardioplegia. In addition to that, there was a statistically significant difference in the maximum values of hs-cTnT and CK.

In summary, St. Thomas 2 cardioplegic solution was associated with significant lower levels of postoperative cardiac biomarkers compared to Bretschneider (Custodiol®) cardioplegic solution and therefore should be recommended in patients undergoing minimal invasive mitral valve repair.

## Figures and Tables

**Table 1:** Patient characteristics.

	Before IPTW				After IPTW			
	Bretschneider (*n* = 123)	St. Thomas (*n* = 61)	Diff	*p*	Bretschneider (*n* = 113)	St. Thomas (*n* = 55)	Diff	*p*
Age (years)	63.0 ± 11.5	64.2 ± 9.8	0.114	0.480	63.3 ± 11.8	64.0 ± 10.2	0.063	0.696
Female	44 (35.8%)	22 (36.1%)	-0.006	0.969	39 (34.3%)	19 (34.0%)	0.007	0.965
Diabetes	3 (2.4%)	1 (1.6%)	0.057	0.728	3 (2.4%)	1 (2.2%)	0.015	0.933
Hypertension	78 (63.4%)	27 (44.3%)	0.391	0.014	66 (58.7%)	31 (56.4%)	0.045	0.791
Hypercholesterolemia	24 (19.5%)	14 (23.0%)	-0.084	0.588	21 (18.7%)	12 (22.7%)	-0.099	0.561
Current smoker	13 (10.6%)	6 (9.8%)	0.024	0.878	11 (9.4%)	5 (9.9%)	-0.016	0.921
BMI	25.3 ± 4.6	25.2 ± 3.8	-0.024	0.884	25.1 ± 3.8	25.5 ± 4.0	0.108	0.519
Preoperative stroke	9 (7.3%)	2 (3.3%)	0.181	0.289	5 (4.6%)	3 (5.4%)	-0.035	0.851
Renal disease	1 (0.8%)	1 (1.6%)	-0.075	0.618	1 (1.0%)	0 (0.0%)	0.141	0.484
Last preoperative creatinine (*μ*mmol/L)	80.5 ± 17.9	84.8 ± 20.7	0.225	0.143	82.0 ± 17.8	80.6 ± 16.1	-0.080	0.622
COPD	3 (2.4%)	3 (4.9%)	-0.132	0.382	3 (2.8%)	1 (2.7%)	0.005	0.977
NYHA III or IV	27 (22.0%)	14 (23.0%)	-0.024	0.878	24 (21.1%)	12 (22.0%)	-0.022	0.896
AF preoperative	24 (19.5%)	12 (19.7%)	-0.004	0.979	23 (20.3%)	12 (21.1%)	-0.019	0.910
Ejection fraction	60.6 ± 7.9	62.0 ± 8.9	0.167	0.277	60.9 ± 7.8	61.4 ± 9.0	0.061	0.717
EuroSCORE 2	1.2 ± 0.8	1.3 ± 0.9	0.054	0.727	1.2 ± 0.8	1.2 ± 0.7	-0.064	0.690
Additive EuroSCORE	3.5 (3.3 to 3.9)	3.8 (3.4 to 4.2)	0.670	0.550	3.5 (3.2 to 3.8)	3.7 (3.3 to 4.2)	0.659	0.589
Logistic EuroSCORE	2.7 (2.4 to 3.0)	2.8 (2.5 to 3.3)	0.586	0.825	2.7 (2.4 to 3.0)	2.8 (2.4 to 3.3)	0.572	0.889

BMI: body mass index; COPD: chronic obstructive pulmonary disease; NYHA: New York Heart Association; AF: atrial fibrillation.

**Table 2:** Perioperative variables.

	Before IPTW				After IPTW			
	Bretschneider (*n* = 123)	St. Thomas (*n* = 61)	Diff	*p*	Bretschneider (*n* = 113)	St. Thomas (*n* = 55)	Diff	*p*
Indication								
Active endocarditis	3 (2.4%)	1 (1.6%)	0.057	0.728	3 (2.2%)	1 (2.2%)	0.003	0.985
Previous endocarditis	5 (4.1%)	1 (1.6%)	0.146	0.399	4 (3.7%)	0 (0.0%)	0.278	0.158
Degenerative	103 (83.7%)	51 (83.6%)	0.004	0.982	97 (85.7%)	44 (80.6%)	0.137	0.437
Calcification	10 (8.1%)	5 (8.2%)	-0.002	0.988	8 (6.7%)	5 (9.1%)	-0.091	0.586
Function	9 (7.3%)	6 (9.8%)	-0.090	0.558	7 (6.1%)	7 (13.1%)	-0.241	0.166
Rheuma	1 (0.8%)	0 (0.0%)	0.128	0.480	1 (0.9%)	0 (0.0%)	0.136	0.484
Ischemia	1 (0.8%)	0 (0.0%)	0.128	0.480	1 (0.8%)	0 (0.0%)	0.126	0.484
Dilatation of annulus	11 (8.9%)	23 (37.7%)	-0.723	0.000	9 (7.6%)	21 (37.6%)	-0.768	0.000
Barlow	15 (12.2%)	16 (26.2%)	-0.362	0.019	13 (11.1%)	14 (25.3%)	-0.374	0.026
Annuloplasty	121 (98.4%)	56 (91.8%)	0.308	0.048	111 (98.6%)	50 (91.1%)	0.345	0.037
Neochordae	95 (77.2%)	48 (78.7%)	-0.035	0.824	89 (78.8%)	42 (76.6%)	0.053	0.763
Resection	4 (3.3%)	1 (1.6%)	0.105	0.534	4 (3.7%)	1 (1.5%)	0.141	0.412
Procedure								
Duration of operation (min)	209.6 ± 35.1	208.0 ± 31.9	-0.048	0.764	207.9 ± 35.0	207.1 ± 31.1	-0.025	0.879
Aortic clamping time (min)	91.2 ± 19.3	89.2 ± 16.0	-0.110	0.495	90.6 ± 19.3	88.4 ± 16.0	-0.124	0.439
Perfusion time (min)	147.8 ± 29.1	143.6 ± 28.4	-0.145	0.356	147.0 ± 29.0	141.8 ± 27.0	-0.188	0.250
Valve size	34.0 ± 3.7	36.8 ± 2.9	0.854	0.000	34.1 ± 3.6	36.6 ± 3.0	0.754	0.000
Severe insufficiency	117 (95.1%)	60 (98.4%)	-0.183	0.303	108 (95.7%)	54 (97.8%)	-0.121	0.526

**Table 3:** Postoperative outcomes: clinical outcomes.

	After IPTW			
	Bretschneider (*n* = 113)	St. Thomas 2 (*n* = 55)	Diff	*p*
30 days mortality	1 (0.7%)	0 (0.0%)	0.118	0.484
Postoperative stroke	0 (0.0%)	1 (1.2%)	-0.155	0.151
MACCE	3 (3.1%)	1 (1.2%)	0.130	0.415
Postoperative MI	3 (2.4%)	0 (0.0%)	0.220	0.321
Postoperative renal failure	0 (0.0%)	1 (1.3%)	-0.164	0.151
Pulmonary infection	4 (3.3%)	0 (0.0%)	0.260	0.223
Sternal infection	1 (0.8%)	0 (0.0%)	0.129	0.484
Intubation > 72 h	1 (1.2%)	0 (0.0%)	0.157	0.484
Reoperation for bleeding	3 (2.7%)	0 (0.0%)	0.235	0.223
Permanent pacemaker	1 (0.7%)	0 (0.0%)	0.118	0.484
AF at discharge	32 (28.5%)	15 (27.7%)	0.018	0.915
Ejection fraction (%)^∗^	50.3 ± 10.3	49.1 ± 14.2	-0.097	0.580
Length of stay (day)	8.5 (8.1 to 8.9)	9.0 (8 to 10)	0.734	0.186

MACCE: major adverse cardiac and cerebrovascular events; MI: myocardial infarction; AF: atrial fibrillation.

**Table 4:** Postoperative biomarkers.

	After IPTW			
	Bretschneider (*n* = 113)	St. Thomas 2 (*n* = 55)	Diff	*p*
First creatinine (*μ*mmol/L)	71.0 ± 19.8	73.8 ± 19.3	0.144	0.381
Max. creatinine (*μ*mmol/L)	86.1 ± 27.5	89.9 ± 27.0	0.138	0.403
First CRP (mg/L)	51.1 ± 26.7	64.2 ± 28.1	0.477	0.005
Max. CRP (mg/L)	163.6 ± 68.0	145.0 ± 81.3	-0.248	0.147
First GFR (mL/min/1.7)	91.5 ± 20.1	88.9 ± 22.8	-0.120	0.479
Max. GFR (mL/min/1.7)	97.1 ± 15.8	92.7 ± 21.4	-0.237	0.173
First potassium (mmol/L)	4.4 ± 0.4	4.6 ± 0.5	0.412	0.017
Max. potassium (mmol/L)	4.6 ± 0.4	4.7 ± 0.5	0.160	0.356
First hemoglobin (g/L)	104.3 ± 16.3	104.1 ± 13.5	-0.011	0.943
Max. hemoglobin (g/L)	118.2 ± 13.9	118.6 ± 16.6	0.028	0.871
First leucocytes (×10^9^/L)	14.1 ± 5.4	13.8 ± 5.8	-0.055	0.744
Max. leucocytes (×10^9^/L)	15.4 ± 5.1	14.7 ± 5.1	-0.142	0.391
First sodium (mmol/L)	139.0 ± 3.5	138.6 ± 2.9	-0.124	0.441
Max. sodium (mmol/L)	141.6 ± 3.9	141.4 ± 2.2	-0.047	0.754
First hs-cTnT (ng/L)	604 (482 to 756)	512 (426 to 616)	0.317	0.052
Max. troponin hs-cTnT (ng/L)	716 (605 to 847)	561 (467 to 674)	0.350	0.047
First CK (U/L)	1186 (1042 to 1351)	1023 (837 to 1250)	0.417	0.103
Max. CK (U/L)	1370 (1222 to 1536)	1152 (972 to 1366)	0.449	0.037
First CKMB (*μ*g/L)	36.5 (32.3 to 41.3)	31.2 (26.6 to 36.7)	0.454	0.059
Max. CKMB (*μ*g/L)	40.0 (35.1 to 45.6)	33.4 (27.3 to 40.8)	0.400	0.295

CRP: C-reactive protein; GFR: glomerular filtration rate; hs-cTnT: high-sensitivity cardiac troponin; CK: creatine kinase; CK-MB: creatine kinase-muscle brain type.

## Data Availability

The data used to support the findings of this study have not been made available because of local ethical guidelines.

## Abbreviations

AF: Atrial fibrillation
BMI: Body mass index
CK: Creatine kinase
CK-MB: Creatine kinase-muscle brain type
COPD: Chronic obstructive pulmonary disease
CRP: C-reactive protein
ECC: Extracorporeal circulation
EF: Ejection fraction
GFR: Glomerular filtration rate
hs-cTnT: High-sensitivity cardiac troponin
ICU: Intensive care unit
IPTW: Inverse probability of treatment weighting
MACCE: Major adverse cardiac and cerebrovascular events
MI: Myocardial infarction
NYHA: New York Heart Association.

## References

[1] Baumgartner H., Falk V., Bax J. J. (2017). 2017 ESC/EACTS guidelines for the management of valvular heart disease. *European Heart Journal*.

[2] Grant S. W., Hickey G. L., Modi P., Hunter S., Akowuah E., Zacharias J. (2019). Propensity-matched analysis of minimally invasive approach versus sternotomy for mitral valve surgery. *Heart*.

[3] Schaefer M., Gebhard M.-M., Gross W. (2019). The efficiency of heart protection with HTK or HTK-N depending on the type of ischemia. *Bioelectrochemistry*.

[4] Buckberg G. D., Athanasuleas C. L. (2016). Cardioplegia: solutions or strategies?. *European Journal of Cardio-Thoracic Surgery*.

[5] Wang Z.-H., An Y., du M. C. (2017). Clinical assessment of histidine-tryptophan-ketoglutarate solution and modified St. Thomas’ solution in pediatric cardiac surgery of tetralogy of Fallot. *Artificial Organs*.

[6] Giordano R., Arcieri L., Cantinotti M. (2016). Custodiol solution and cold blood cardioplegia in arterial switch operation: retrospective analysis in a single center. *The Thoracic and Cardiovascular Surgeon*.

[7] Hummel B. W., Buss R. W., DiGiorgi P. L. (2016). Myocardial protection and financial considerations of custodiol cardioplegia in minimally invasive and open valve surgery. *Innovations: Technology and Techniques in Cardiothoracic and Vascular Surgery*.

[8] Mkalaluh S., Szczechowicz M., Dib B. (2018). Early and long-term results of minimally invasive mitral valve surgery through a right mini-thoracotomy approach: a retrospective propensity-score matched analysis. *PeerJ*.

[9] Matzelle S. J., Murphy M. J., Weightman W. M., Gibbs N. M., Edelman J. J. B., Passage J. (2014). Minimally invasive mitral valve surgery using single dose antegrade custodiol cardioplegia. *Heart Lung and Circulation*.

[10] Chambers D. J., Haire K., Morley N. (1996). St. Thomas’ hospital cardioplegia: enhanced protection with exogenous creatine phosphate. *The Annals of Thoracic Surgery*.

[11] Bretschneider H. (1980). Myocardial protection. *The Thoracic and Cardiovascular Surgeon*.

[12] Gebhard M., Preusse C., Schnabel P., Bretschneider H. (1984). Different effects of cardioplegic solution HTK during single or intermittent administration. *The Thoracic and Cardiovascular Surgeon*.

[13] Gallandat Huet R., Karliczek G., Homan van der Heide J. (1988). Clinical effect of Bretschneider-HTK and St. Thomas cardioplegia on hemodynamic performance after bypass measured using an automatic datalogging database system. *The Thoracic and Cardiovascular Surgeon*.

